# Nck adapter proteins: functional versatility in T cells

**DOI:** 10.1186/1478-811X-7-1

**Published:** 2009-02-02

**Authors:** Marcus Lettau, Jennifer Pieper, Ottmar Janssen

**Affiliations:** 1 University Hospital Schleswig-Holstein Campus Kiel, Institute of Immunology, Molecular Immunology, Arnold-Heller-Str 3, Bldg 17, D-24105 Kiel, Germany

## Abstract

Nck is a ubiquitously expressed adapter protein that is almost exclusively built of one SH2 domain and three SH3 domains. The two isoproteins of Nck are functionally redundant in many aspects and differ in only few amino acids that are mostly located in the linker regions between the interaction modules. Nck proteins connect receptor and non-receptor tyrosine kinases to the machinery of actin reorganisation. Thereby, Nck regulates activation-dependent processes during cell polarisation and migration and plays a crucial role in the signal transduction of a variety of receptors including for instance PDGF-, HGF-, VEGF- and Ephrin receptors. In most cases, the SH2 domain mediates binding to the phosphorylated receptor or associated phosphoproteins, while SH3 domain interactions lead to the formation of larger protein complexes. In T lymphocytes, Nck plays a pivotal role in the T cell receptor (TCR)-induced reorganisation of the actin cytoskeleton and the formation of the immunological synapse. However, in this context, two different mechanisms and adapter complexes are discussed. In the first scenario, dependent on an activation-induced conformational change in the CD3ε subunits, a direct binding of Nck to components of the TCR/CD3 complex was shown. In the second scenario, Nck is recruited to the TCR complex via phosphorylated Slp76, another central constituent of the membrane proximal activation complex. Over the past years, a large number of putative Nck interactors have been identified in different cellular systems that point to diverse additional functions of the adapter protein, e.g. in the control of gene expression and proliferation.

## The Nck family of adapter proteins

**Figure 1:**
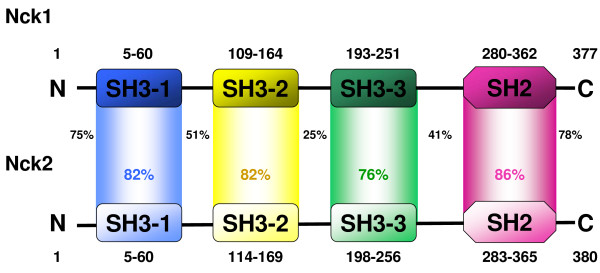
**Modular composition of Nck adapter proteins**. Nck adapters are proteins of 47 kDa that are built of three SH3 domains and a C-terminal SH2 domain linked by small spacer regions. Nck1 displays 68% amino acid identity to Nck2. As indicated in the figure, the differences are mainly located in the linker regions between the interaction modules, whereas the individual SH2 and SH3 domains show a high degree of homology. Modular domains of Nck1 (NP_006140) and Nck2 (AAH07195) have been assigned using the simple modular architecture research tool SMART . The sequence homology between the interaction modules and the linker regions was determined using the SIM alignment tool for protein sequences . Percent values indicate the degree of identity of the respective regions.

Nck (non-catalytic region of tyrosine kinase) proteins are adapter proteins of 47 kDa that are almost exclusively built of one SH2 domain and three SH3 domains (Fig. 1) [1]. In human cells, the Nck family comprises two members (Nck1/Nckα and Nck2/Nckβ, also termed Grb4). The human nck1 gene has been localised to the locus 3q21 of chromosome 3 and the nck2 gene to 2q12 of chromosome 2. Nck1 and Nck2 display 68% identity at the amino acid level. Notably, the largest differences are mainly located in the linker regions between the interaction modules. Moreover, Nck1 and Nck2 are to some extent functionally redundant and neither Nck1 nor Nck2 knock-out mice exhibit an apparent phenotype whereas double knock-out mice die in utero [2]. Nevertheless, some studies provided evidence for non-overlapping functions of Nck1 and Nck2 in certain cell types, including for example an exclusive regulation of actin polymerization in response to platelet-derived growth factor (PDGF) and epidermal growth factor (EGF) treatment by Nck2 in fibroblasts and breast carcinoma cells (MTLn3) [3,4]. Moreover, the SH2 domain of Nck2 but not of Nck1 interacts with the docking protein Disabled-1 [5]. In terms of more general functions, only Nck2 has been implicated in the control of neuritogenesis [6]. However, hardly any Nck1- or Nck2-specific downstream target has been identified so far. In fact, in many instances the interactions have not been clearly attributed to Nck1 or Nck2. Mostly, interactions proposed for one Nck variant have not been tested with the respective other isoprotein. In essence, the published data are somewhat inconsistent regarding the question as to whether Nck1 and Nck2 binding partners overlap or rather diverge. Therefore, in the following, Nck1 and Nck2 are generally termed Nck, but readers should keep in mind that the described functions/interaction partners are not necessarily attributed to both isoproteins. Systematic studies are still needed to shed light on common or distinct binding partners and functions of Nck1 and Nck2.

## Nck interaction partners and functional implications

Being a prototypic adapter protein, the modular architecture of Nck allows for numerous protein-protein interactions. Over the past years, more than 60 interaction partners have been described in different cellular systems (listed in tables 1 and 2 and reviewed in [1,7]). This short review does not discuss all potential interactions but rather highlights some selected examples to point to the diversity of processes that Nck adapters are involved in.

**Table 1:** SH2 domain interaction partners of Nck

**Protein**	**Reference**
A36R viral protein	[131]

Ack	[132]

Bcr-Abl	[109]

β-Dystroglycan	[133]

BLNK	[134]

Cas-L	[135]

Caveolin-2	[136]

Disabled-1	[5]

Dok1	[137]

Dok2	[138,139]

EGFR	[8]

EphB1 (receptor for Ephrin B1)	[17]

Ephrin B1 (EphB1 ligand)	[27]

FAK	[116]

Git1	[22]

Git2	[23]

HGFR	[16]

IRS-1	[140]

IRS-3	[141]

Nephrin	[24,25]

P130Cas	[142]
PDGFR	[8]

RET	[143]

Slp76	[26]

SOCS3	[144]

Tir (bacterial protein)	[145]

TrkB	[146]

VEGFR1	[11]

VEGFR2	[10]

**Table 2:** SH3 domain interaction partners of Nck

**Protein**	**Reference**
Abl	[107]

Ack1	[108]

ADAM15	[33]

Bcr-Abl	[109]

Casein kinase 1 (γ2)	[110]

Cbl	[26,111]

CD3ε	[34]

DCC	[112]

DOCK180	[113]

Dopamine D4 receptor	[114]

Dynamin	[101]

eIF2β	[105]

FAK	[115,116]

FasL	[90,91]

GC-NAP	[117]

hnRNPκ	[118]

HPK1	[119]

IRS-1	[120]

NAP1BP	[121]

NckAP1	[122]

NIK	[123]

N-WASP	[47]

PAK1	[73]

PAK3	[124]

PINCH	[125]

PRK2	[126]

RalGPS	[127]

R-Ras	[102]

Sam68	[95]

SOCS7	[93]

Sos	[99]

Synaptojanin	[118]

TBK1	[128]

TNIK	[129]

WASP	[30]

WIP	[31]

YAP65	[130]

Only in a few studies, interactions have been mapped to individual SH3 domains. In fact, several studies do not attribute a given interaction to an individual SH3 domain. Moreover, SH3 domains which do not contribute to a direct interaction might well influence binding in a cooperative manner. Thus, the reader is encouraged to consult the cited literature for further information on the Nck interacting protein of interest.

### SH2 domain

Src homology 2 (SH2) domains are modules that comprise about 100 amino acids and interact with phosphorylated tyrosine residues. Specificity is guided by the amino acids surrounding the phosphotyrosine (pY). Over the past years, Nck was shown to bind to several tyrosine-phosphorylated proteins via its SH2 domain. Thus, Nck isoproteins associate with activated receptor-tyrosine kinases such as the EGF receptor (EGFR) [8,9], vascular endothelial growth factor receptor (VEGFR) [10-13], PDGF receptor (PDGFR) [3,14,15], hepatocyte growth factor receptor (HGFR) [16], and with the Ephrin receptor EphB1 [17,18]. Also via its SH2 domain, Nck may associate with Dok (downstream of kinase signaling) proteins which seem to play a negative regulatory role in tyrosine kinase signaling [19-21]. Moreover, Nck interacts with Git1 (G protein-coupled receptor kinase interactor) [22] and Git2 [23], two ADP ribosylation factor GTPase activating proteins (Arf GAPs) that are functionally associated with cell attachment, spreading and motility. Via binding to phosphorylated nephrin, Nck coordinates cytoskeletal dynamics to establish intercellular junctional architecture in kidney podocytes [24,25]. The SH2-mediated interaction with Slp76 (SH2 domain-containing leukocyte protein of 76 kDa) [26] seems prerequisite for the important adapter function of Nck in T cells (see below).

It was suggested that the SH2 domains of Nck1 and Nck2 differ with respect to their binding properties, especially since some non-overlapping functions have been observed [3,4,6]. In case of the PDGFR, the phospho-tyrosine residue pY751 was reported to be Nck1-specific [14], whereas pY1009 was Nck2-specific [3]. Moreover, only Nck2 associates with tyrosine-phosphorylated Disabled-1, an important adapter protein involved in brain cell positioning during development [5]. Likewise, phosphorylated ephrin B1 (the ligand for EphB1) seems to associate with the SH2 domain of Nck2 but not with the Nck1 SH2 domain [27]. However, recent reports providing structural insight into the Nck1 and Nck2 SH2 domains indicate that both binding modules are more or less indistinguishable with respect to their binding specificities and both recognize the consensus motif pYDxV(AYST)x(DEC) [22]. The few differences in ligand binding described so far might therefore rely on other parts of the Nck molecule. Similar intramolecular interactions that modulate SH2-mediated target recognition have been found to alter the affinity between Gads/Slp76 and LAT [28]. In addition, it was also speculated that SH2 interactions may be modulated by adjacent SH3 domains and/or by the variable linker loops that connect the individual binding domains in a given protein. However, such intramolecular effects would probably only mildly affect the overall pattern of binding partners of Nck1 and Nck2, but rather modulate the affinity of a given interaction.

### SH3 domains

SH3 domains are globular modules of about 50–60 amino acids which mediate a rather constitutive binding to proline-rich motifs in corresponding target proteins. In many cases, interaction partners for individual SH3 domains have been identified, e.g. by pull down assays. Also for the individual SH3 domains of Nck, several associated proteins have been named (Table 2). Notably, in such assays, the identified interaction partners exhibit a clear preference for individual SH3 domains [1,7]. Thus, the observation that several Nck ligands bind to more than one SH3 domain of Nck suggests that a cooperative interaction is necessary for tight complex formation [29]. Nck utilizes the specificity of its individual SH3 domains to facilitate multiple interactions with different binding partners. Many of these binding partners are functionally associated with the regulation of the actin cytoskeleton including for example the (neuronal) Wiskott-Aldrich Syndrome protein ((N-)WASP) [30] and the WASP interacting protein (WIP) [31]. It is well established that the multidomain adapter protein WASP activates the Arp2/3 (actin-related proteins 2/3) complex that finally induces the formation of branched actin filament networks [32]. Thus, Nck links receptor-induced activation signals to key regulators of the actin cytoskeleton.

Other interaction partners including for example the Son of Sevenless homologue (Sos) and the Src-activated during mitosis protein (Sam68) indicate distinct roles of Nck in the control of cellular signaling, gene expression and proliferation [1,7]. Moreover, Nck binding to certain splice variants of the "a disintegrin and metalloprotease" ADAM15 points to a role of Nck in malignancy since these variants are selectively increased in breast cancer cells [33]. The association of the SH3-1 domain with a proline-rich stretch in the CD3ε subunit of the T cell receptor (TCR) once more points to an important role of Nck in TCR signaling (see below) [34].

Recent studies of the structural properties of individual SH3 domains provided more insight into the ligand binding preferences and specificities of the Nck1-1 (being the first and most N-terminal SH3 domain of Nck1), Nck1-2, Nck2-2 and Nck2-3 SH3 domains [35,36]. Although the two analyzed SH3 domains of Nck1 adopt the five-stranded β-barrel fold typical of SH3 domains, they differ with respect to the electrostatic potentials of their surfaces. Whereas the Nck1-2 SH3 domain possesses a neutral and a highly negatively charged region (and thus resembles the Nck2-3 SH3 domain in this respect), the Nck1-1 SH3 domain exhibits a significantly weaker negative charge [35,36]. The structures of the Nck1-3 and the Nck2-1 (insoluble) SH3 domain have not been solved at high resolution yet. However, the data obtained so far clearly underscore the functional relatedness of the two isoproteins and the individual properties of the single SH3 domains that account for the observed differences in ligand binding.

Interestingly, only very recently it was described that Nck might also associate with the inactive form of the dsRNA-activated protein kinase PKR (see below). However, this novel type of interaction seems to be independent of the Src homology domains and thus offers a first indication for further potential protein-protein interactions of Nck [37].

## Nck and T cell effector function

### Actin reorganisation in T cell activation

T cells play a central role in adaptive immunity by enhancing or suppressing immune responses through cytokine secretion or by eliminating virus-infected or transformed cells. T cell activation and effector function is tightly controlled and relies on fairly stable cell-cell contacts especially during primary activation and communication with antigen-presenting cells (APCs). Once a T cell encounters its specific antigen on an APC in the lymph nodes or spleen in an appropriate MHC context, it rapidly reorients its cellular organelles to the contact area in a process accompanied by complex structural and cytoskeletal changes. The primary antigenic stimulation ultimately results in cell cycle progression and clonal expansion. The T cells leave the lymphoid tissue and search the periphery for infected or transformed cells carrying their cognate antigen. Upon recognition of a target cell, the T cell again reorients its cellular content to the intercellular contact zone. In this case, the secondary stimulation results in a polarized secretion of meanwhile matured cytolytic granules and/or cytokines into the organized intracellular cleft (reviewed in [38,39]). Despite the complex architecture of the established cellular contacts, cell-mediated cytotoxicity is a highly dynamic process. A single T cell can eliminate multiple targets consecutively, rapidly rearrange established contacts and even form stimulatory and lytic synapses simultaneously [40,41]. Obviously, several independent but coordinated cellular processes contribute to T cell activation and effector function. These include an integrin-mediated adhesion and contact stabilisation, the formation of an immunological synapse (IS) with defined central and peripheral signaling platforms, and the establishment of a threshold-dependent cell polarity for the directed secretion of cytokines and lytic granules. It is clear that all these processes are strictly dependent on rapid dynamic changes of the lymphocyte cytoskeleton. Accordingly, engagement of the TCR activates multiple actin-regulatory proteins that work in concert to drive actin polymerization at the IS [39,42].

At the molecular level, TCR ligation results in the activation of the T cell-specific Src-type kinases Fyn and Lck which phosphorylate the crucial immunoreceptor tyrosine-based activation motifs (ITAMs) within the TCR-associated CD3-chains to serve as docking-sites for the two SH2 domains of the Syk-type kinase ZAP70 (zeta chain-associated protein of 70 kDa). Activated ZAP70 phosphorylates an array of key regulators of the membrane-proximal activation complex including the linker for the activation of T cells (LAT). As a transmembrane adapter protein, LAT couples upstream signaling of Lck/ZAP70 to downstream signaling events including calcium flux, phosphatidylinositol turnover and Ras activation. The adapter protein Gads (Grb2-like adapter downstream of Shc) binds phosphorylated LAT and subsequently recruits the scaffold protein Slp76 to the activation complex [43-45]. Upon phosphorylation by ZAP70, Slp76 binds the crucial guanine nucleotide exchange factor Vav and Vav in turn activates the Rho family GTPases Cdc42 and Rac. Rho family GTPases consist of Rac, Cdc42 and Rho, small G proteins activated by GEFs such as Vav and localized to the cell membrane by prenylation. In their activated form, Rho GTPases facilitate the regulation of actin filament formation through effector proteins such as actin-related proteins 2/3 (Arp2/3) and WASP family members (reviewed in [39,42]). The WASP family includes five proteins: WASP, N-WASP, WAVE1, WAVE2 and WAVE3. The expression of WASP is restricted to hematopoietic tissues whereas N-WASP and WAVE 2 are ubiquitously expressed. WAVE1 and WAVE3 are enriched in the brain but are also expressed throughout the mammalian body. The main function of the multidomain adapter protein WASP is the activation of the Arp2/3 complex that finally leads to the formation of branched actin filament networks. WASP is controlled by autoinhibition and is activated by binding to Cdc42 via its GTPase-binding domain. Activity can be further enhanced by phosphoinositides binding to a basic region of the WASP molecule. Interactions with proteins like WIP, intersectin or Grb2 and also phosphorylation by Src kinases have also been reported to affect WASP activity. However, in most cases, the precise molecular mechanisms are only poorly understood [32,46]. Since Nck not only passively interacts with WASP and could thus recruit WASP to molecular activation clusters [30], but also somehow modulates WASP activity [47], it was believed that this adapter protein also plays an essential role in the regulated activation-dependent reorganization of TCR-associated signaling complexes and platforms.

### Nck and TCR signaling: Slp76 and/or CD3ε

**Figure 2:**
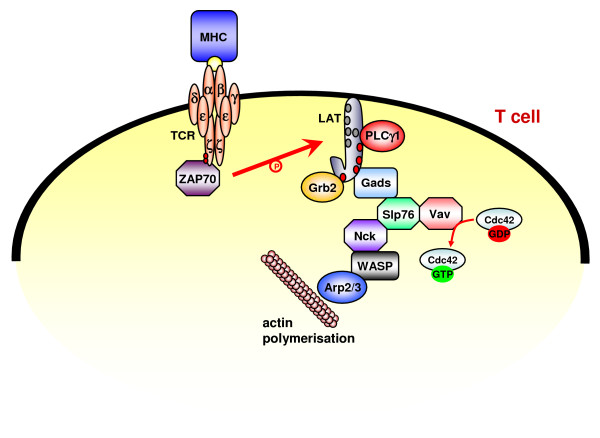
**TCR-induced actin-reorganization: Nck binding to phosphorylated Slp76**. T cell activation is initiated by antigen-presenting cells (APCs) containing stimulatory MHC-peptide complexes. Src family protein tyrosine kinases mediate phosphorylation of TCR associated ITAMs thereby creating docking sites for the Syk-type kinase ZAP70. After activation by Src kinases, ZAP70 phosphorylates LAT. LAT contains nine tyrosine residues which, when phosphorylated, act as docking sites for adaptor proteins such as Grb2 and Gads. Slp76 is recruited to the membrane-proximal activation complex through its interaction with the SH3 domains of LAT-associated Gads. Phosphorylated Slp76 associates with the SH2 domain of Nck. Nck then recruits the multidomain adapter protein WASP. The GEF Vav, which is also recruited by Slp76, promotes the GTP-loading of the small Rho-GTPase Cdc42 that is critically involved in WASP activation. WASP then activates the Arp2/3 complex that initiates the formation of branched actin filament networks.

Although Nck is unanimously regarded as a linker between the TCR and the cytoskeleton, it is still a matter of debate, how exactly Nck associates with activation clusters around the TCR/CD3 complex. As mentioned, Nck has been shown to bind phosphorylated Slp76 via its SH2 domain and to recruit WASP via SH3-mediated interactions. In this scenario, Slp76 functions as a scaffold bringing Nck and WASP into proximity with Vav1 and Cdc42-GTP (Fig. 2) [26,48,49]. Of note, the involved proteins do not necessarily need to be present in a single complex. Instead separate tools may exist in the cells, e.g. distinct molecules of Slp76 may associate with either Vav or Nck.

Gil and coworkers, however, proposed that Nck directly binds to a proline-rich sequence (PRS) within the CD3ε chain that only gets available due to a conformational change upon TCR ligation. This association is mediated by the SH3-1 domain and it precedes tyrosine phosphorylation. The stable overexpression of Nck SH3-1-EGFP negatively modulated cell spreading, IL-2-release and synapse formation/maturation in Jurkat cells, presumably by preventing the association of endogenous Nck with CD3ε. However, in this experimental setting, the overexpressed SH3-1 domain may also block the interaction of Nck with other binding partners besides CD3ε. Moreover, transduction of peripheral blood mononuclear cells with an antibody binding near the CD3ε-PRS (mAb APA1.1) decreased proliferation after TCR ligation compared to control-transduced cells, at least underscoring the role of the CD3ε-PRS. The authors proposed that the recruitment of Nck and associated regulatory proteins such as WASP, WIP or Pak1 to CD3ε displays an alternative means to link T cell activation to the cytoskeleton independent of preceding tyrosine phosphorylation [34]. However, the functional relevance of this interaction was questioned when Barda-Saad and colleagues showed that the TCR-induced tyrosine phosphorylation of LAT and Slp76 is indispensable for the recruitment of Nck and WASP to induce actin polymerization [48].

In addition, in a retrogenic approach, T cells expressing CD3ε with a mutated proline-rich sequence (PRS) on a CD3ε-null background developed normally. Although the binding of Nck to CD3ε was completely abrogated, there was no apparent defect in positive or negative selection. Furthermore, the proliferative response of T cells to staphylococcal enterotoxin B and to anti-CD3 mAb was normal, indicating that the interaction of CD3ε with Nck (and/or other SH3 domain containing proteins) might not be essential for T cell development and T cell responses to strong antigens [50]. However, it was more recently shown that TCR crosslinking and the conformational change in the CD3ε subunit seem to be required for full tyrosine phosphorylation of different downstream effectors [51] and that the conformational change is also transmitted to the cytoplasmic tails of the other CD3 subunits that close up to form a compact structure that allows for Nck binding [52].

In an earlier study, Risueno and colleagues could demonstrate that the conformational change within CD3ε is also elicited *in vivo *in the lymph nodes of mice after antigen exposure. Employing the mAb APA1.1, that recognizes an epitope within the activation-dependently exposed region of CD3ε, they further showed that the conformational change depends on the strength of the used agonist [53]. Moreover, using this mAb, the conformational change could be clearly observed in situ in double-positive thymocytes. These were predominantly located in the cortex and the corticomedullar junction in close contact with epithelial and dendritic cells indicating a specific role of the CD3ε cytoplasmic tail in thymocyte selection [54].

In another experimental setting using naïve and differentiated murine CD8^+ ^T cells, strong MHC (major histocompatibility complex) agonists triggered cytokine release in both cell types whereas weak agonists, only affected differentiated cells. In this scenario, the responsiveness correlated with the ability of the agonist to elicit a CD3ε conformational change as measured by the ability of CD3ε to bind Nck. The mutation of the CD3ε proline motif and the ITAM significantly impaired the response to weak antigens in differentiated cells [55]. Interestingly, in this context, Nck binds to an uncommon PxxDY motif that encompasses the ITAM of CD3ε [56] and proline point mutations abrogate CD3ε ITAM phosphorylation [55]. This might indicate that the CD3ε motif serves to amplify low-avidity TCR signals by promoting ITAM phosphorylation and subsequently protein kinase C Θ (PKCΘ) recruitment and synapse formation in at least certain clonotypes of differentiated CD8^+ ^T cells. Thus, this mechanism could contribute to the higher relative sensitivity of effector T cells.

Notably, Src kinase (Lck)-mediated phosphorylation of the tyrosine residue (Y166), which is shared by the ITAM and the PxxDY motif, seems to be required for the recruitment of the tandem SH2 cassettes of ZAP70, which in turn forms one of the most crucial early events in T cell activation. Conversely, tyrosine phosphorylation of the PxxDY motif abolishes Nck binding, indicating that Y166 might serve as a molecular switch to determine whether CD3ε is competent for SH3 or SH2 binding. This, however, would suggest that Nck binding to CD3ε might rather be transient and lost before ZAP70 binding takes place [56]. Therefore, in a hypothetical model, the binding of Nck to the exposed PxxDY via its first SH3 domain allows the recruitment of other signaling molecules containing proline-rich sequences or phosphotyrosine residues via the second and/or third SH3 or the SH2 domain, respectively. In turn, the phosphorylation of CD3ε would at the same time facilitate the binding of ZAP70 and the dissociation of Nck [55].

In strong contrast, another report indicates that Nck binding to PxxDY might rather inhibit a subsequent phosphorylation of Y166 by Fyn and Lck [57]. However, this was concluded from the structure of the Nck2 SH3-1/CD3ε complex and experimentally demonstrated by the ability of the SH3-1 domain of Nck2 to block the tyrosine phosphorylation of a CD3ε peptide (aa 143–183) by recombinant Fyn (and Lck) in an *in vitro *phosphorylation assay. Moreover, in this study, Nck has been implicated in negative modulation of TCR surface expression [57]. Accordingly, the PxxDY motif encompasses a putative internalization motif (YxxI/L), albeit this sequence has been described to behave only as a weak internalization signal in the first report of CD3ε endocytosis [58]. Nevertheless, overexpression of Nck led to a decrease in TCR surface expression while overexpression of a Nck SH3-1 mutant affected TCR surface expression to a lesser degree [57].

**Figure 3:**
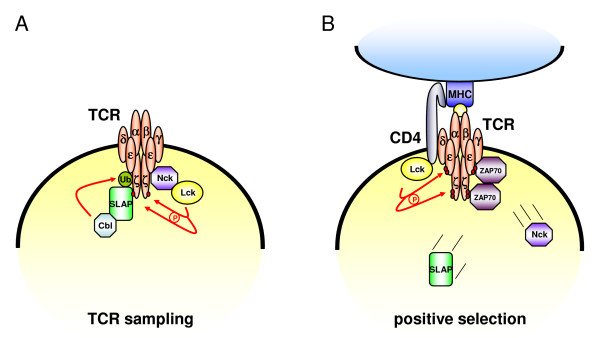
**Suggested model for the function of the CD3ε PRS in double-positive thymocytes**. **(A) **Nck associates with CD3ε in the absence of pMHC and recruits Lck, which phosphorylates TCRζ. Subsequently, phosphorylated TCRζ recruits SLAP, and SLAP-associated c-Cbl ubiquitinates (Ub) TCRζ. This leads to the degradation of TCRζ and thus increases the TCR sampling rate. **(B) **pMHC-mediated ligation of the TCR results in more complete phosphorylation of the CD3 ITAMS. In this context, the interaction of CD3ε-bound Nck with Lck may be necessary to prime the system and allow CD4 coreceptor-dependent Lck to initiate a full activation response with phosphorylation of the CD3ε ITAMS. This results in the recruitment of ZAP70, the dissociation of Nck and SLAP and thus the stabilization of TCR surface expression. (Figure accords with models suggested in [59]).

A recent study employing a highly sophisticated mouse model clarified some of the conflicting results described before [59]. The authors established a knock-in mouse where the critical CD3ε motif was replaced by another sequence naturally occupying an analogous sequence in the cytoplasmic tail of FcεR1γ to assure an unaltered distance of the CD3ε ITAM from the membrane and the neighbouring CD3 subunits. Surprisingly, they observed a constitutive association of the first Nck SH3 domain with CD3ε in freshly isolated thymocytes and mature T cells. This association was, however, further enhanced upon stimulation. Moreover, TCR surface expression in double-positive thymocytes was increased due to a reduced SLAP- (Src-like adapter protein-) dependent degradation of CD3ζ. In double-positive thymocytes, activated Lck initiates a signaling cascade that involves phosphorylation of the CD3ζ subunit of the TCR complex and results in the recruitment of SLAP. SLAP functions as an adapter to target the E3 ubiquitin ligase activity of c-Cbl to phosphorylated CD3ζ chains present in the fully assembled TCR for degradation [60-64]. As a consequence, fewer TCR complexes recycle back to the surface and TCR expression on double-positive thymocytes is thus significantly lower than on mature T cells [65]. Thus, during preselection of double-positive cells, the CD3ε PRS might recruit Lck via Nck to control the phosphorylation of nearby CD3ζ subunits and SLAP-dependent degradation of the TCR. However, a direct interaction of Nck with Lck has not been demonstrated yet. On the other hand, the CD3ε^ΔPRS/ΔPRS ^double-positive cells showed a decreased responsiveness to weak self pMHC agonists compared to wild-type double-positive cells, indicating that the CD3ε PRS enhances the signaling capability, an effect also observed in previous studies [53,55]. Also in this study, the CD3ε PRS was dispensable for the response to strong antigens. The authors suggest that by associating with the CD3ε PRS, Lck molecules would be pre-coupled to the TCR complex and might thus confer higher signaling competence to weak antigens whereas the PRS is dispensable when stimulated with strong antigens. Such a model would at least in part explain the observed ability of double-positive cells to respond to weak antigens. Thus, the CD3ε PRS obviously negatively modulates the sensitivity of double-positive cells by downregulating cell surface TCR and at the same time enhances their sensitivity to weak antigens. The authors suggest an appealing explanation for this in the first view apparent discrepancy taking into account the peculiar features of double-positive thymocytes during T cell development (Fig. 3). CD4^+ ^CD8^+^double-positive cells undergo TCRα gene rearrangements and only a few double-positive cells that express an αβ TCR capable of low-affinity interactions with self peptides bound to MHC molecules mature into single-positive cells during positive selection. Double-positive cells that fail to recognize self pMHC complexes undergo apoptosis as well as double- and single-positive cells that bind self-antigens with high affinity. Physiologically, the CD3ε PRS and SLAP may act together to decrease the pool of cycling TCRs present in double-positive cells and thus increase the sampling rate of new TCRα chains that are sequentially synthesized. However, to permit positive selection of some of the TCR complexes that are present on the cell surface with low copy number, the CD3ε concurrently increases the signaling output (Fig. 3A). Finally, positive selection and subsequent ITAM phosphorylation might allow binding of ZAP70 thereby replacing Nck and SLAP and preventing further SLAP/CD3ε PRS-dependent degradation of TCR/CD3 complexes (Fig. 3B). This would result in the rapid upregulation of TCR surface expression associated with the transition of double-positive cells to single-positive thymocytes. However, such a potentially elegant mechanism has not yet been experimentally confirmed in detail.

### Nck and p21-activated kinases (PAKs)

Upon TCR ligation, two p21-activated kinases (PAK1 and PAK2) are activated [66-69]. In non-lymphoid cells, PAK has been linked to various events associated with cytoskeletal dynamics [70]. PAK activation contributes to TCR-induced Erk activation, calcium flux and the NFAT transcriptional response [67-69], although the precise mechanism of PAK activation in T cells is still elusive. Clearly, PAK requires Lck and ZAP70 for activation [67,71]. Moreover, the interaction with the active forms of the Rho GTPases Rac1 and Cdc42 also facilitates PAK activation. Several lines of evidence suggest that Nck recruits PAK to the plasma membrane in response to growth factors including EGF and PDGF. In this scenario, Nck associates with autophosphorylated tyrosine kinase receptors via its SH2 domain and binds to the first proline-rich region at the N-terminus of PAK via its second SH3 domain [72,73]. In T cells, a pathway involving LAT, Slp76, Nck and Vav was suggested to mediate recruitment and activation of PAK [49]. However, there is also evidence for an alternative pathway, since a PAK mutant (not able to interact with Nck) can still be activated [71]. PAK also interacts with the guanine nucleotide exchange factor Pix (Pak-interacting exchange factor) and the Arf GAP Git in a trimolecular complex [71]. Pix has been identified as a GEF for Rac1 and Cdc42 [74,75]. Pix itself interacts with the Git family proteins Git1 and Git2 which in turn interact with multiple other proteins including focal adhesion complex proteins as FAK (focal adhesion kinase) and paxillin [76,77]. This PAK1/Pix/Git1 complex is rapidly recruited to the T cell/APC contact site independent of Slp76 and Vav1. Moreover, this complex may be crucial for PAK activation by recruiting it to the immunological synapse. Altered membrane localization and thus an increase in local concentration has been shown to be crucial for PAK activation [78,79]. In this scenario, the PAK/Pix/Git complex is presumably recruited to the IS via integrins, as Git interacts with paxillin, an important adapter protein in integrin signaling [80]. Of note, since PAK activation initially also requires Lck and ZAP70 but within the PAK/Pix/Git complex seems to be independent of Nck, Slp76 and LAT, this may represent a very early event in TCR signaling. As the PAK/Pix/Git complex translocates to the site of intercellular contact within 1–3 minutes [81], PAK activation might thus be involved in the early phase of initial contact and target cell recognition. However, as recent data indicate that Nck interacts with Git1 [22] and Git2 [23], Nck might be also involved in the LAT, SLp76 and Vav-independent activation of PAK.

### Nck and SLAM-associated protein (SAP)

Costimulatory immunoreceptors of the SLAM (signaling lymphocyte activation molecule) family are functionally associated with TH2 cell priming, memory B cell generation, antibody production, activation of natural killer (NK) cells and NKT cell development. They mediate their effects through interactions with members of the SAP (Slam-associated protein) family (reviewed in [82]). SAP mediates the recruitment of the Src kinase Fyn to SLAM. SAP is a small cytosolic protein composed of a single SH2 domain and a 28 aa C-terminal tail. Deletion or mutation of SAP causes the X-linked lymphoproliferative syndrome characterized by reduced NK and CTL activity as well as decreased B cell function and impaired NKT cell development. Interestingly, the SAP SH2 domain binds to the SH3 domain of Fyn and simultaneously to Y281 of SLAM in a phosphorylation-independent manner. This not only recruits Fyn to SLAM, but also activates its kinase activity enabling the intense phosphorylation of SLAM, the recruitment of further downstream proteins and thus propagates signaling [82-86]. Recently, Nck has been shown to interact with SAP via its second SH3 domain, but affinity was greatly enhanced when the third SH3 domain was also present. Interestingly, SAP depletion attenuated Slp76 and Nck1 phosphorylation whereas SAP overexpression enhanced Slp76 phosphorylation. In accordance with the well-established role of Nck in initiating activation-induced actin reorganisation in T cells, the depletion of SAP resulted in decreased actin polymerization. Moreover, SAP depletion was also accompanied by a decrease in LAT phosphorylation, Erk activation and cell proliferation, highlighting the role of SAP in T cell activation. Of note, the exact role of its interaction with Nck1 in this context has to be elucidated. Similar to Nck, also SAP interacts with Pix [87] and could thus also regulate the formation of a PAK/Pix/Nck complex.

### Nck and FasL

**Figure 4:**
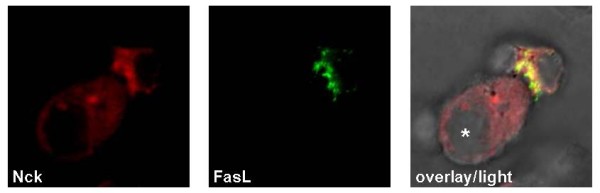
**Subcellular localization of Nck and the death factor FasL in conjugates of Jurkat T cells and EBV-transformed B-LCL**. Upon target cell recognition the death factor FasL is transported to the cytotoxic immunological synapse where it colocalizes with Nck. Jurkat T cells were transiently transfected with FasL, cocultured with superantigen-pulsed B-LCL (*, B lymphoblastoid cell line)), fixed, permeabilized and stained for FasL with anti-FasL mAb NOK-1 and respective AlexaFluor488-conjugated secondary antibodies and for Nck with an anti-Nck pAb and corresponding AlexaFluor546-conjugated secondary reagents.

Nck has also been functionally associated with the death factor Fas ligand (FasL). The FasL is a type-2-transmembrane protein belonging to the tumor necrosis factor (TNF) family of death factors. In cytotoxic T and NK cells, FasL is stored in association with so-called secretory lysosomes to avoid unwanted damage. Only upon recognition of a target cell, these vesicles are transported to the site of intercellular contact, thus releasing cytotoxic molecules into the synapse and exposing FasL locally on the plasma membrane [88,89]. Nck interacts with an extended proline-rich stretch within the cytoplasmic part of the FasL via its second and third SH3 domain [90,91] and is critically involved in the recruitment of FasL and/or its storage granules to the cytotoxic immunological synapse (Fig. 4) [90].

## Other aspects of Nck biology: nuclear localization of Nck, cell-cycle arrest and inhibition of translation

Only very recently, Nck has been described to translocate to the nucleus upon cellular stress [92]. In this scenario, the Nck-interacting protein SOCS7 [93,94] (that in contrast to Nck contains a NLS/NES) regulates the nucleocytoplasmic distribution of Nck. Septins in turn bind to SOCS7 and this interaction retains both SOCS7 and Nck in the cytoplasm. Following DNA damage, both proteins accumulate in the nucleus. This accumulation is essential for morphological changes (e.g. the disintegration of stress fibers and loss of cell-polarity) and for the activation of downstream members of the DNA damage cascade and cell-cycle arrest. Thus, Nck somehow links a DNA damage checkpoint to the actin cytoskeleton [92]. In this scenario, the depletion of Nck from the cytosol presumably accounts for the observed effects rather than a specific action within the nucleus. Nevertheless, it has been shown, that the array of nuclear binding partners significantly differs from cytosolic interactions [95]. However, only few nuclear Nck-interacting proteins have been described so far.

Regarding such nuclear binding partners, Nck apparently interacts with Sam68 [95]. Sam68 (Src activated during mitosis) belongs to the STAR (signal transducers and activators of RNA) family of RNA binding proteins (reviewed in [96]). Sam68 has been functionally associated with several aspects of RNA metabolism including (regulated) splicing, regulation of RNA stability and RNA transport/localization [96]. It contains several proline-rich stretches enabling interactions with SH3 domains. Furthermore, the C-terminal domain contains several tyrosines that are subject to phosphorylation by a variety of tyrosine kinases and then serve as docking sites for SH2 domains. Both, the association with binding partners and the observed tyrosine phosphorylation might negatively modulate the RNA binding capability of Sam68 [96]. Furthermore, Sam68 contains two nuclear localization sequences in its C-terminal part and the nuclear localization of Sam68 seems to be regulated by arginine methylation. Whereas hypomethylated Sam68 is located in the cytoplasm, the methylated form is predominantly found in the nucleus [97,98]. Thus, Sam68 is an appealing molecule for transducing information from signaling pathways to the RNA machinery.

Interactions of Nck with Grb2, Sos [99-101] and R-Ras [102] implicate that Nck might also be involved in Ras activation and thus in cell proliferation in general. Moreover, Nck participates in the cellular responses to ER stress such as the inhibition of translation. In this context, Nck is integrated into signaling pathways regulating eIF2α (eukaryotic initiation factor 2α) phosphorylation [103,104], providing a common mechanism to downregulate protein synthesis in stressed cells. Upon overexpression, Nck increases protein translation [105] and impairs eIF2α phosphorylation and thus stress-induced attenuation of translation [37]. Mechanistically, Nck directly interacts with the β-subunit of the initiation factor eIF2 via its first and third SH3 domains [105] and participates in the assembly of a complex containing the serine/threonine protein phosphatase 1c (PP1c) and thus promotes eIF2α dephosphorylation [103,106]. Moreover, Nck complexes with the eIF2α kinase PKR and thus interferes with PKR activity. Again, the precise molecular mechanism of this inhibition is not yet clear. Taken together, Nck adapters are not only employed in various cell conditions but also by different cellular compartments to generate or modulate specific cellular responses.

## Conclusion

Over the past years, many different interaction partners of Nck adapter proteins have been described (as summarized in Tables 1 and 2[107-146]). Accordingly, Nck has been associated with a plethora of diverse processes including for instance cellular activation, motility, and effector function but also axon guidance and neuritogenesis, glomerular filtration barrier in the kidney, responses to DNA damage and cell stress and the development of mesodermal structures during development. Obviously, Nck plays an important role in the T cell compartment, participating in different and interdependent pathways of T cell activation and effector function during different stages of T cell selection and maturation. In agreement with its functional versatility, the double knock-out of the Nck adapters Nck1 and Nck2 in mice results in early embryonic lethality, whereas single knock-out mice have no apparent phenotype. Although this indicates a functional redundancy of the two isoproteins, some non-overlapping functions have been described. Thus, systematic studies are pending in order to clarify to which extent Nck isoprotein interaction partners and functions overlap or diverge in a given cellular system.

## Abbreviations

ADAM: a disintegrin and metalloprotease; APC: antigen-presenting cell; Arf: ADP ribosylation factor; Arp2/3: actin-related proteins 2/3; Cbl: Casitas B-lineage lymphoma; CTL: cytotoxic T lymphocyte; Dok: downstream of tyrosine kinase; EGF(R): epidermal growth factor (receptor); eIF: eukaryotic initiation factor; EphB1: ephrin receptor B1; FasL: Fas ligand; FcεR1γ, Fc: (fragment, crystalizable)-epsilon receptor 1 gamma chain; GAP: GTPase-activating protein; GEF: GDP exchange factor; Git: G protein-coupled receptor kinase interactor; Grb2: growth factor receptor-bound protein 2; HGF(R): hepatocyte growth factor (receptor); ITAM: immunoreceptor tyrosine-based activation motif; LAT: linker of activated T cells; Lck: leukocyte-specific protein tyrosine kinase; mAb: monoclonal antibody; (p)MHC: (peptide-loaded) major histocompatibility complex; Nck: non-catalytic region of tyrosine kinase; NES: nuclear export signal; NFAT: nuclear factor of activated T cells; NK: natural killer; NLS: nuclear localization signal; PAK: p21-activated kinase; PDGF(R): platelet-derived growth factor (receptor); Pix: PAK-interacting exchange factor; PKC: protein kinase C; PKR: protein kinase RNA-activated; PRS: proline-rich sequence; Sam68: Src-activated during mitosis, 68 kDa; SAP: SLAM-associated protein; SH: Src homology; SLAM: signaling lymphocyte activation molecule; SLAP: Src-like adapter protein; Slp76: SH2 domain-containing leukocyte protein of 76 kDa; SOCS: suppressor of cytokine signaling; Sos: son of sevenless; TCR: T cell receptor; (N-)WASP: (neuronal) Wiskott-Aldrich syndrome protein; WAVE: WASP family verprolin homologous; WIP: WASP-interacting protein; ZAP70: zeta chain-associated protein of 70 kDa.

## Competing interests

The authors declare that they have no competing interests.

## Authors' contributions

ML designed and wrote the manuscript and provided original data presented as figures. JP contributed to the discussion of Nck interaction partners and provided figures. OJ critically supervised the work throughout the writing process. All authors have read and approved the final manuscript.
